# Number and mode of inheritance of QTL influencing backfat thickness on SSC2p in Sino-European pig pedigrees

**DOI:** 10.1186/1297-9686-43-11

**Published:** 2011-03-06

**Authors:** Flavie Tortereau, Hélène Gilbert, Henri CM Heuven, Jean-Pierre Bidanel, Martien AM Groenen, Juliette Riquet

**Affiliations:** 1 INRA, UMR 0444 Génétique Cellulaire, F-31326 Castanet-Tolosan, France; 2 INRA, UMR 1313 Génétique Animale et Biologie Intégrative, F-78352 Jouy-en-Josas, France; 3 Wageningen University, Animal Breeding and Genetics Group, 6700 AH Wageningen, The Netherlands

## Abstract

**Background:**

In the pig, multiple QTL associated with growth and fatness traits have been mapped to chromosome 2 (SSC2) and among these, at least one shows paternal expression due to the IGF2-intron3-G3072A substitution. Previously published results on the position and imprinting status of this QTL disagree between analyses from French and Dutch F2 crossbred pig populations obtained with the same breeds (Meishan crossed with Large White or Landrace).

**Methods:**

To study the role of paternal and maternal alleles at the IGF2 locus and to test the hypothesis of a second QTL affecting backfat thickness on the short arm of SSC2 (SSC2p), a QTL mapping analysis was carried out on a combined pedigree including both the French and Dutch F2 populations, on the progeny of F1 males that were heterozygous (A/G) and homozygous (G/G) at the IGF2 locus. Simulations were performed to clarify the relations between the two QTL and to understand to what extent they can explain the discrepancies previously reported.

**Results:**

The QTL analyses showed the segregation of at least two QTL on chromosome 2 in both pedigrees, i.e. the IGF2 locus and a second QTL segregating at least in the G/G F1 males and located between positions 30 and 51 cM. Statistical analyses highlighted that the maternally inherited allele at the IGF2 locus had a significant effect but simulation studies showed that this is probably a spurious effect due to the segregation of the second QTL.

**Conclusions:**

Our results show that two QTL on SSC2p affect backfat thickness. Differences in the pedigree structures and in the number of heterozygous females at the IGF2 locus result in different imprinting statuses in the two pedigrees studied. The spurious effect observed when a maternally allele is present at the IGF2 locus, is in fact due to the presence of a second closely located QTL. This work confirms that pig chromosome 2 is a major region associated with fattening traits.

## Introduction

Many QTL associated with economically important traits like growth, fatness and meat quality have been detected since the 2000 s, as reviewed by Bidanel and Rotschild in 2002 [1]. However, even for those that have been fine-mapped, successful identification of the causal mutation is rare. In 1999, a paternally expressed QTL affecting backfat thickness (BFT) and muscle mass was identified on the short arm of SSC2 close to the IGF2 gene in crosses between Large White (LW) and European Wild Boar [2] and between LW and Pietrain [3]. In 2003, Van Laere et al. [4] reported that the IGF2-intron3-G3072A substitution is the causal mutation. This mutation affects the binding site of a repressor and up-regulates IGF2 expression in skeletal muscles and heart, inducing major maternally imprinted effects on muscle growth, heart size and fat deposition. Therefore, selection for animals carrying allele A at this locus is a major issue in pig production. Analysis of the frequency and effects of this mutation in pig populations of different genetic origins showed that both wild (G) and mutant (A) alleles still segregate in modern populations (LW × Pietrain cross) [5,6], and that allele A is very rare or even nonexistent in local breeds and Wild Boars [7]. The strong favourable effect of allele A was confirmed in both Spanish [8] and Polish [9] LW and Landrace (LR) breeds. In 2004, Jungerius et al. [5] demonstrated that the mutation also explains the major imprinted QTL for backfat thickness in a cross between Meishan (MS) and European White pigs (LW and LR). Yet, although significant effects of the IGF2 mutation were revealed both by ultrasonic and carcass BFT measurements, the presence of a second QTL at a position near 40 cM, as previously described in this population by de Koning [10], cannot be excluded [5]. In the French LW × MS cross, QTL affecting loin weight and BFT on carcass have also been detected near the IGF2 locus [11]. However, surprisingly, no imprinting effect could be detected [12], although the breeds involved are similar (European White breeds and MS) in the Dutch and French studies, and the MS animals in both crosses are related. It has been shown that spurious imprinting effects can exist because of maternal effects [13] or because of linkage disequilibrium [14]. The aims of the present work were to estimate more precisely the IGF2 substitution effect by combining the two MS × European intercrosses, and to investigate further the genetic determinism of the SSC2p chromosomal region by testing the hypothesis of an additional QTL segregating on SSC2 in these populations. In addition, simulation studies were conducted to investigate how the presence of two QTL could affect the apparent mode of inheritance of IGF2 alleles.

## Methods

### Animals and phenotypic data

The French and Dutch F2 MS × European breeds crosses and the recorded phenotypes have been described previously [15-17]. Briefly, the French INRA-PORQTL pedigree consisted of 12 F0 (six LW sires and six MS dams), 26 F1 (six sires and 20 dams) and 521 castrated male F2 pigs. All animals were born and raised at the INRA GEPA experimental research unit (Surgères, Charentes). The Dutch pedigree, obtained from the University of Wageningen (WU), was initiated by mating 19 MS sires to 126 LW and LR dams, resulting in an F1 population of 39 sires and 265 F1 dams, which produced a total of 1212 F2 offspring. The Dutch pedigree was bred in five different breeding companies. Among the 39 Dutch half-sib families, only the 24 largest (more than 30 progeny) were retained in the present analysis in order to homogenize the family structure of the two pedigrees.

Among the traits recorded in the two populations, BFT measured between the third and the fourth rib of carcass at 6 cm from the spine [10,11] was considered here as the main common trait shared in both designs affected by the QTL under study. This trait was recorded on 565 Dutch pigs (castrated males and females) and on 521 French pigs (castrated males only).

Phenotypic data were first adjusted for fixed effects and covariates with the GLM procedure in SAS^® ^(SAS^® ^9.1, SAS^® ^Institute, Inc.). The models used to adjust the data included the effects of batch, slaughter day and carcass weight for the INRA pedigree and breeding company, sex, slaughter day and carcass weight for the Dutch pedigree.

### Genetic data

Animals from both pedigrees were genotyped for 11 microsatellites evenly spaced on chromosome 2 (SW2443 (0 cM); SWC9 (2 cM); SW2623 (11 cM); SW256 (23 cM); S0141 (37 cM); SW240 (51 cM); S0091 (76 cM); S0010 (90 cM); S0368 (96 cM); S0378 (108 cM) and S0036 (149 cM)), as previously reported [18].

Genotyping of the IGF2-intron3-G3072A substitution was performed on some of the F0 and F1 animals of both pedigrees. Previously, F1 boars and their parents [5] from the Dutch pedigree had been genotyped by the pyrosequencing technique (Pyrosequencing AB) described in [4]. In the French design, all F0 and F1 animals were genotyped by PCR-RFLP using primers 5'-GGACCGAGCCAGGGACGAGCCT-3' and 5'-GGGAGGTCCCAGAAAAAGTC-3'. The polymerase chain reaction was carried out at 57°C using the GC-RICH PCR system (ROCHE), in presence of 1 M GC-RICH Resolution solution, and 1.5 mM of MgCl2. PCR-RFLP with the restriction enzyme ApeK1 was used to detect the mutation according to the manufacturer's recommendations for time, temperature and buffer conditions. Then, genotypes of all F2 animals at the mutation were inferred for non recombinant haplotypes inherited from F1 individuals, using information from the pedigree and from the transmission of parental haplotypes for surrounding markers (SW2443 and SWC9). No genotype was assigned for recombinant F2 piglets with a heterozygous A/G parent or if the mother had not been genotyped for the mutation. The parental origin of the allele inherited at the A/G substitution was also inferred when possible according to the phase they inherited from their parents.

### QTL analyses

QTL detection was performed on the adjusted data using the QTLMap software [19,20] as explained in [18]. Parameter estimates were obtained by maximization of the likelihood with a Newton-Raphson algorithm, and a Likelihood Ratio Test (LRT) was computed at each cM along SSC2. The maximum LRT along the linkage group indicated the most likely position for a QTL. For each sire, the substitution effect corresponds to the difference between the Meishan and the European alleles, a positive effect indicating an increased value of the trait due to Meishan alleles. The average QTL substitution effect was computed as the mean of the absolute values of the sire substitution effects. QTL significance thresholds were empirically computed using 1000 simulations under the null hypothesis, assuming an infinitesimal polygenic model for the trait, as described by Gilbert and Le Roy [21].

QTL detection analyses were carried out first for the French and Dutch pedigrees separately, and then for the combined pedigree. A potential second QTL segregating within these pedigrees was investigated with two different methods. First, the multi-QTL option of QTLMAP was used to detect two linked QTL on SSC2 for BFT. The alternative hypothesis (H1) of two QTL segregating was compared to the null hypothesis of one QTL segregating at the IGF2 locus. The LRT were computed following a grid-search strategy, using 5 cM steps along the chromosome. Significance thresholds were empirically estimated by 1000 simulations under the null hypothesis, as described by Gilbert and Le Roy [21]. In a second analysis, the segregation of a potential additional QTL was investigated: (1) by analysing the data from the progeny of sires homozygous at the IGF2 locus (G/G) and (2) by performing a QTL detection analysis on the full combined pedigree with a model that included IGF2 as a fixed effect.

### Mode of inheritance of the QTL

Analyses of variance (ANOVA) were carried out to infer the inheritance pattern of the SSC2 QTL, using data adjusted for the previously described fixed effects. Tests were applied to compare different effects *¿_i _*in the model *Y_ij _*= *¿ *+ *¿_i _*+ *¿_ij_*, where *Y_ij _*is the adjusted performance of individual *j *of genotype *i *(see below), *¿ *is the population mean, *¿_ij _*is the residual error of individual *j *of genotype *i*, and *¿_i _*is the tested effect. Three different effects for *¿_i _*were built based on the following inheritance patterns:

- Only the paternally inherited allele at the mutation has an effect (model IGF2pat, *i *= {A,G})

- Only the maternally inherited allele at the mutation has an effect (model IGF2mat, *i *= {A,G})

- Both the paternally inherited allele and the maternally inherited allele at the mutation have effects (model IGF2patmat, *i *= {AA,AG,GA,GG}, the paternal allele being written first).

These analyses of variance were applied to all F2 individuals of the combined pedigree, of both pedigrees separately, and to sub-groups of animals defined according to the genotype of the parents at the IGF2 mutation:

- F2 having A/G sires

- F2 having A/G dams

- F2 having A/G sires and G/G dams

- F2 having G/G sires and A/G dams

- F2 having A/G sires and A/G dams.

### Detection of spurious effects of the maternally inherited IGF2-allele

Simulation studies were performed with the QTLMap software to evaluate the power of the inheritance pattern analyses and of the additional QTL studies proposed in this paper, in the presence of a major imprinted gene in the chromosomal region investigated. A QTL segregating at 44 cM affecting the trait was simulated while assuming a paternal effect of the IGF2 locus. Only the phenotypes were simulated; family structures and genotypes were obtained from real data from the two pedigrees. The effect of the IGF2pat model was set to 0.48 phenotypic standard deviations of the trait (as estimated in the data set, see below). The QTL was assumed to be bi-allelic, with the Q allele decreasing backfat level as compared to the q allele. The simulated QTL effect represented the substitution effect of allele q by allele Q. Simulations were then performed with the following parameter values:

- Frequency of the QTL alleles:

- in the F0 European breeds (French F0 males and Dutch F0 females) for allele Q: 0.25, 0.50, 0.75 or 1.00

- in the Meishan populations (French F0 females and Dutch F0 males) for allele q: 1.00. Fixation was assumed based on the small size of the original population and based on the fact that MS individuals were also homozygous for IGF2.

- Effect of the simulated QTL: 0.22 or 0.32 or 0.42 phenotypic standard deviations of the trait.

For each simulation, a QTL analysis was performed as described above and the value of the maximum LRT (LRTmax) and its position were recorded. Simulated phenotypes were exported to the SAS^® ^software and analyses of variance were performed as previously described to determine which inheritance pattern was detected depending on the simulated parameters, applying either the IGF2pat or the IGF2mat models. For the analyses of the two pedigrees separately, 2000 replicate simulations were performed for each combination of frequency × effect parameters. For the combined pedigree, 2000 replicates were performed with an effect of 0.32 and a frequency of 0.50 for the QTL in both grandparental populations, as the two pedigrees were reciprocal. The percentage of replicates returning significant results for each pattern of inheritance of IGF2 and detection of the QTL were then computed from the 2000 replicates for each situation.

## Results

### Genotyping results for the IGF2 mutation

The IGF2-intron3-G3072A mutation was genotyped for most of the F0 and F1 founders of both pedigrees (Table 1). Presence of IGF2 wild type and mutant alleles in the Dutch pedigree was reported previously [5]. To summarize, all MS F0 sires were homozygous (G/G) for the wild allele, and allelic heterogeneity was identified for the LW F0 dams: in the two Dutch LR lines, all the dams were homozygous (G/G), whereas in the three other LW lines allele A was found with frequencies over 80%. Among the 24 sire families selected for our study, 12 F1 sires were homozygous (G/G), and 12 F1 sires were heterozygous (A/G). These 24 F1 sire families involved 65 heterozygous females (A/G) and 71 homozygous females (G/G), while the genotype of 38 F1 dams remained unknown.

**Table 1:** Distribution of genotypes at IGF2-intron3-G3072-A substitution

	Dutch pedigree					French pedigree				
	
	A/A	A/G	G/G	unknown	total	A/A	A/G	G/G	unknown	total
F0 males	0	0	19	0	19	0	5	1	0	6
F0 females	30	21	22	27	100	0	0	6	0	6
F1 males	0	12	12	0	24	0	2	4	0	6
F1 females	0	65	71	38	174	0	15	5	0	20
F2	342	180	20	23	565	226	188	22	85	521

Numbers of heterozygous F2 are given regardless parental origin of alleles.

All F0 and F1 animals were genotyped in the French pedigree (Table 1). All MS F0 dams were homozygous G/G. Among the six LW sires, five were heterozygous (A/G) and one was homozygous for the mutant allele (A/A). Among the six F1 sires, four were homozygous (G/G) and two were heterozygous (A/G). These six F1 sire families involved 15 heterozygous females (A/G) and five homozygous females (G/G).

The genotypes of the F2 pigs at the IGF2-intron3-G3072A were inferred from the genotypes of their parents at the mutation and the haplotypes inherited at the surrounding SW2443 and SWC9 microsatellite markers. A complete genotype at the IGF2 mutation could be obtained for 90% of the F2 pigs. Analyses of variance to test the inheritance pattern of the IGF2 mutation were thus performed on 980 F2 animals (543 Dutch F2 and 437 French F2). For ANOVA studies with the IGF2patmat model, the heterozygous (A/G) F2 pigs were split into two groups depending on the parental origin of the two alleles. For the combined pedigree, the total numbers of animals of each genotype at the mutation were 44 A/A, 568 G/G, 155 A/G and 213 G/A, with the first allele identifying the paternal allele.

### QTL detection

First, each pedigree was analysed independently. In the French pedigree, the maximum of the test statistic was obtained in the IGF2 region (0 cM) but was only significant at the 10% threshold. Analysis of the Dutch pedigree gave a significant result at the 5% threshold, but the maximum of the test statistics was reached at 28 cM.

**Figure 1:**
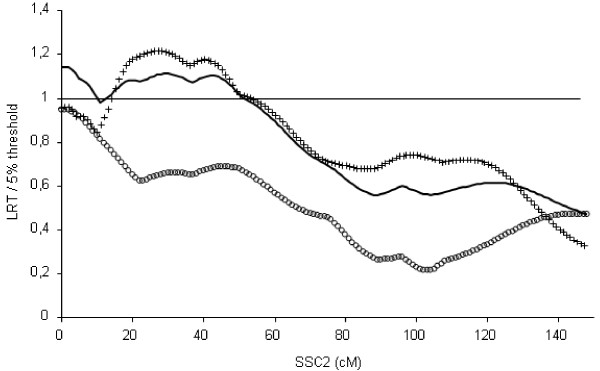
**QTL detection on SSC2 in the three studied pedigrees**. Solid, circled and crossed lines represent respectively the combined, French and Dutch pedigrees; for each analysis, the LRT is presented as a proportion of the 5% threshold on the chromosome.

The QTL detection analysis was then performed on the combined pedigree (Figure 1). The maximum LRT value was obtained in the region surrounding the IGF2 position. However, between 13 and 51 cM, the values of the test statistics were also higher than the 5% threshold.

A multi-QTL analysis was then performed with the combined pedigree but neither significant nor suggestive results were obtained for the hypothesis of two QTL segregating within both pedigrees.

**Figure 2:**
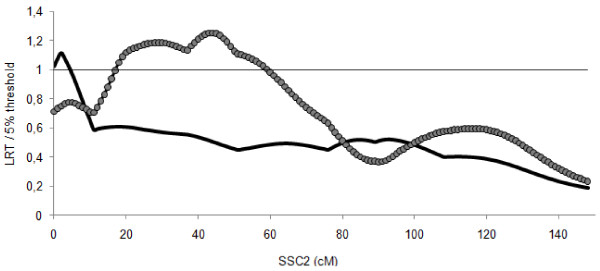
**QTL analyses on SSC2 on sub-groups of the combined pedigree**. The solid line represents the QTL detection results from the segregating sire families (A/G sires) and the circles-marked line results from the no-segregating sire families (G/G sires) at the IGF2-intron3-G3072A mutation; for each analysis, the LRT is presented as a proportion of the 5% threshold on the chromosome.

The QTL detection analysis performed on the 14 families from sires heterozygous at the mutation revealed a significant QTL close to the IGF2 locus (Figure 2). The decrease of the test statistic values downstream from the IGF2 gene was abrupt and no other region reached the 5% threshold. A complementary analysis was performed on the 16 families originating from homozygous F1 sires (G/G) and detected a significant QTL at 44 cM. The substitution effects estimated at this second QTL position showed that, among the 16 sires analysed, three F1 sires could not be validated as heterozygous for the QTL. The 13 remaining sires were heterozygous with MS alleles associated with high BFT values in nine families and with low BFT values in two families. For the two remaining sires, the breed origin of the favourable allele could not be determined. On average, the QTL effect was estimated to be 0.32 s.d. of the trait. A similar result was obtained with the combined pedigree using phenotypic data corrected for the effect of the IGF2-intron3-G3072A genotype (data not shown). These results clearly indicate that a significant QTL affecting BFT is segregating around 40 cM on SSC2.

### Mode of inheritance of the QTL

Analyses of variance were performed on different sub-groups of animals to investigate the effect of the IGF2 mutation. Results obtained using the IGF2pat model confirmed the strong effect of the paternally inherited allele at the mutation (Table 2), since significant p-values were observed in all studied sub-groups of F2 pigs. The p-values obtained with the IGF2patmat model were always very similar to those obtained with the IGF2pat model (data not shown). To investigate the potential effect of the maternally inherited IGF2 allele, the IGF2mat model was also tested. When the analysis was performed on the F2 progeny of heterozygous dams, a significant p-value was obtained with the combined pedigree (p = 0.04). When the analysis was carried out on the progeny of heterozygous dams mated to homozygous sires, a significant p-value was also observed (p = 0.01). Analysing each pedigree independently, results tended to be significant (p < 0.10) for these two progeny sub-groups in the French pedigree and for the F2 produced from A/G dams and G/G sires in the Dutch pedigree (Table 2).

**Table 2:** Statistical analyses of inheritance of the IGF2 mutation effect

F2 studied	Model	Combined pedigree	French pedigree	Dutch pedigree
All	IGF2 pat	<0.0001 ***	<0.0001 ***	<0.0001 ***
All	IGF2mat	0.30	0.15	0.95
From A/G F1 sires	IGF2pat	<0.0001 ***	0.0007 ***	<0.0001 ***
From A/G F1 sires	IGF2mat	0.94	0.91	0.69
From A/G F1 dams	IGF2pat	0.0001 ***	0.005 **	0.005 **
From A/G F1 dams	IGF2mat	0.04 *	**0.08 ¿**	0.26
From A/G sires and G/G dams	IGF2pat	<0.0001 ***	0.01 *	0.001 ***
From A/G sires and G/G dams	IGF2mat	-	-	-
From G/G sires and A/G dams	IGF2pat	-	-	-
From G/G sires and A/G dams	IGF2mat	0.01 *	**0.07 ¿**	**0.08 ¿**
From A/G sires and A/G dams	IGF2pat	0.0004 ***	0.02 *	0.008 **
From A/G sires and A/G dams	IGF2mat	0.88	0.93	0.97

ANOVA were done on BFT standardized residuals with either the allele inherited at IGF2 from the sire (pat) or from the dam (mat); *** p-value < 0.001, ** p-value <0.01, * p-value < 0.05, ¿ p-value < 0.10; in bold are indicated the p-values < 0.10 obtained with the maternal allelic effect model

### Detection of spurious effects of the maternally inherited IGF2-allele

**Figure 3:**
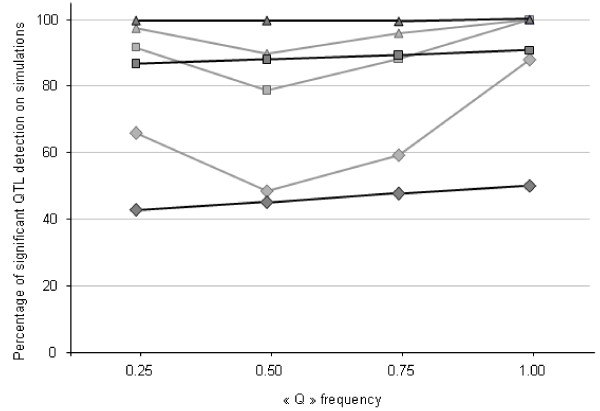
**Percentages of replicates for which the simulated QTL was detected, depending on the frequency of the Q allele simulated in the European grand-parental populations**. The QTL was considered detected when the 5% threshold was reached; analyses were performed for each pedigree independently (in grey for the French design, in black for the Dutch design) and for three values for the QTL effect (diamonds 0.22 s.d., squares 0.32 s.d. and triangles 0.42 s.d.).

The simulated QTL was detected in about 80% of replicates when its effect was at least 0.32 s.d. regardless of the frequency of allele Q in the European grand-parental population (Figure 3). When the simulated QTL had a small effect (0.22), the French pedigree tended to be more powerful than the Dutch pedigree to detect the QTL. With the Dutch design, the simulated QTL was detected in fewer than 50% of replicates. For the simulations performed with the combined pedigree, the QTL was detected in 88% of replicates.

**Figure 4:**
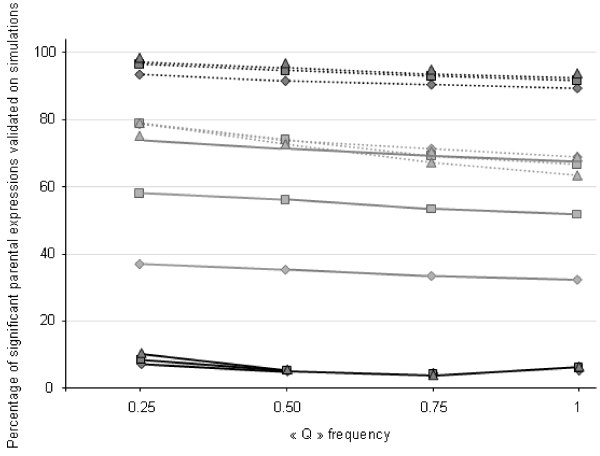
**Percentage of replicates that resulted in a statistically significant effect for the allele inherited from the sire or from the dam at the IGF2 mutation**. The effects were considered significant when the p-value was lower than 5%; the dotted and solid lines represent, respectively, the results obtained for the allele inherited from the sire and from the dam at the IGF2 mutation; different frequencies of the Q allele in the European grand-parental populations were tested; analyses were performed for each pedigree independently (in grey for the French design, in black for the Dutch design) and for three values of the QTL effect (diamonds 0.22 s.d., squares 0.32 s.d. and triangles 0.42 s.d.).

ANOVA was first carried out with the IGF2pat model, using all families. For both pedigrees, the simulated effect of the paternally inherited allele at IGF2 was detected in most replicates (Figure 4). The Dutch pedigree gave more significant results than the French pedigree. When the frequency of the simulated Q allele increased in the European populations, the percentage of replicates resulting in a significant effect for the paternally inherited allele decreased. With the combined pedigree, 83% of replicates showed a significant effect of IGF2 on backfat thickness.

Using the model of maternal inheritance on simulated imprinted paternally expressed IGF2 effects, the proportion of results reaching significance for an effect of the maternal allele at IGF2 (IGF2mat) was expected to be low or null. Variance analyses were performed on the sub-group of progeny produced by heterozygous dams regardless of the genotypes of the sires. With the Dutch pedigree, few replicates led to validation of the maternal expression. In contrast, with the French pedigree, more significant results were obtained (Figure 4). When the simulated QTL had a large effect (0.42 s.d.) and a low frequency of the Q allele was simulated in the European F0 (0.25), up to 75% of the replicates gave a significant result for the IGF2mat model in the French pedigree.

With the combined pedigree, 6.6% of the simulations detected a significant maternally inherited allele effect. When only progeny from the G/G sires among the heterozygous dams' families were considered, the effect of the allele inherited from the mother at the IGF2 mutation was significant in 23% of replicates.

## Discussion

The aim of this study was to confirm the existence of two QTL associated with BFT on SSC2p and to further dissect the imprinting effect of the IGF2 gene, in order to resolve contradictory results published for the French and Dutch MS × European pedigrees [5,12]. The two designs were based on similar founder breeds (MS were crossed with LW and/or LR) and they contributed equally (considering the number of F2) to the so-called "combined pedigree". However, two major differences should be noted. First, the two pedigrees are reciprocal: the MS breed was used as the sire breed in the Dutch pedigree and as the dam breed in the French one. The second discrepancy lies in the pedigree structure: a limited number of large F1 sire families were produced in the French pedigree, whereas the Dutch pedigree consisted in many small F1 sire families.

Our objectives were to (1) show that another QTL on SSC2p is associated with BFT in addition to the IGF2 gene and (2) determine the most likely hypothesis to explain the discrepancies regarding observed imprinting effects in the two pedigrees. Two hypotheses were proposed: a) the imprinting of IGF2 gene is not complete and b) the mendelian effect detected at 0 cM (at the position of the IGF2 locus) in the French pedigree [12] is in fact an artefact due to genetic linkage between IGF2 and a second QTL.

### Detection of QTL underlying BFT on the short arm of SSC2

Analysis of the combined pedigree with the single-QTL model gave a most likely position of the QTL at 0 cM, but LRT values over the 5% threshold were obtained for all positions in the first 50 cM of SSC2 (Figure 1). When the two pedigrees were analysed independently, two different most likely QTL positions were obtained, in accordance with previously published results on the two pedigrees, i.e. at 0 cM in the French pedigree [11,17] and in an interval between positions 20 and 50 cM in the Dutch pedigree [22,23]. After genotyping the Dutch pedigree for the IGF2-intron3-G3072A mutation, it was concluded that the IGF2 QTN, localised at 0 cM, explained most of the observed paternally expressed QTL for BFT on SSC2 [5]. However, the presence of an additional QTL around 30 cM could not be excluded. In the present work, nearly every position on the short arm of SSC2 was significant, which is consistent with the large variability of positions found in the Dutch pedigree, and tends to confirm the hypothesis that more than one QTL associated with BFT is segregating in this region. However, this hypothesis had not been validated before for these two pedigrees.

With the combined pedigree, the multiple-QTL model gave no significant result, so that the hypothesis of two co-segregating QTL could not be validated. These results indicate that the F1 sires in general are not heterozygous for both loci and/or that the dataset does not provide enough information to validate the alternative hypothesis, which might be due to the proportion of homozygous sires for the IGF2 mutation in the combined pedigree (0.5).

The separate analyses of the A/G and G/G F1 fathers provided evidence for the segregation of two QTL, the IGF2 mutation and a second one most likely positioned at 44 cM, which is close to the position initially reported in the Dutch pedigree [10]. Segregation of a QTL affecting BFT around 40 cM was also reported by Lee et al. [24] in a Wild Boar × MS pedigree in which all founders were A/A for the IGF2-intron3-G3072A mutation. Therefore, our results confirm the hypothesis of a second QTL segregating in the Dutch LW × MS cross, as suggested by Jungerius et al. [5], and extend this observation to the French cross.

### Is the IGF2 gene only paternally expressed?

The IGF2 gene has been studied in detail in several species and, in most cases, paternal expression has been described. Nevertheless, modifications of the imprinted status of genes have been reported in humans (most often associated with diseases) [25]. For the IGF2 gene, such pathological modifications have already been described [26,27]. However, a study on the loss of imprinting of the IGF2 gene in colorectal cancers has also shown a loss of imprinting in normal mucosa and peripheral blood leukocytes [28]. Moreover, Sakatani et al. [29] have reported maternal expression of IGF2 in a healthy human population. Li et al. [30] have demonstrated that IGF2 P1 transcripts are bi-allelically expressed in all studied organs from adult healthy pigs. In addition, several studies have reported that the imprinting pattern of a locus can be variable over ontogenetic time [31] or under different environmental effects [32].

One present objective was to evaluate whether the differences of the imprinting status previously reported for the French [12] and the Dutch [5] pedigrees could be clarified. Significant effects of the allele inherited from the dam at the IGF2 mutation were obtained when analysing the progeny of A/G mothers, which is not in accordance with exclusive paternal expression of the IGF2 gene. Simulations showed that segregation of a second QTL at 40 cM from IGF2 can lead to the false detection of expression of the maternally inherited IGF2-allele in the French pedigree, whereas this was almost never observed in simulations of the Dutch or the combined pedigree. The discrepancies between simulation results can be explained by differences in the pedigree structures. The Dutch pedigree is based on 104 matings between F0 animals that produced many small half- and full-sib families (24 F1 sires and 174 dams), whereas the French pedigree is based on six matings only between the F0 animals that produced six large half- and full-sib families (six sires and 20 dams). Another difference between the two designs was the allele frequencies at the IGF2-intron3-G3072A mutation: in the French design, 75% of the F1 dams were heterozygous at the mutation whereas only 47% dams where heterozygous in the Dutch design. Since the simulations were performed using the real genotype data at the IGF2-intron3-G3072A mutation, we could not estimate the influence of the percentage of heterogeneous dams. The relative impact of the different family designs and allele frequencies at the mutation could not be differentiated with these simulations. Nevertheless, the ANOVA and simulation analyses suggest that the differences in the effect of the maternal allele at the IGF2 locus can be caused by the segregation of an additional QTL at 44 cM. The genetic linkage between IGF2 and a second QTL with an effect of 0.32 s.d. is high enough to create this artefactual maternal effect, even if the two loci are relatively distant.

## Conclusion

Since 2003, several studies have reported the effect of the IGF2-intron3-G3072A mutation on BFT. Besides this QTN, several studies tend to show that additional loci in the surrounding chromosomal region could influence the same trait. By combining two F2 designs, this study demonstrates that a second significant QTL affecting pig fatness is localised around 44 cM and that segregation of this second locus can explain the maternal effect that was observed in the French pedigree at the IGF2 locus. Thus, selection schemes against BFT should not only take the status at the IGF2 mutation into account but also genotypes at other QTL in the region.

QTL for other economically important traits have been described on SSC2p, including QTL affecting daily feed intake [33] and teat number [34]. For these traits, the influence of the IGF2-intron3-G3072A mutation via pleiotropic effects has been excluded [5,33]. Therefore, the short arm of SSC2 seems to be an important chromosomal region for pig production. Thus, fine-mapping the other QTL on this chromosome will be of major interest. This task will require a cautious design of fine-mapping experiments since the pedigree structures and the variety of loci in the region can lead to false conclusions.

## Competing interests

The authors declare that they have no competing interests.

## Authors' contributions

FT carried out the QTL detection and statistical analyses. HG participated in those analyses. HCMH provided the information and the phenotypic data sets of the Dutch pedigree. JPB provided the information and the phenotypic datasets of the French pedigree. MAMG and JR conceived the study. JR participated in its design and coordination and helped to draft the manuscript. All authors contributed and approved the final manuscript.
